# Cryopreservation of virus: a novel biotechnology for long-term preservation of virus in shoot tips

**DOI:** 10.1186/s13007-018-0312-9

**Published:** 2018-06-12

**Authors:** Min-Rui Wang, Wen Yang, Lei Zhao, Jing-Wei Li, Ke Liu, Jing-Wei Yu, Yun-Feng Wu, Qiao-Chun Wang

**Affiliations:** 1 State Key Laboratory of Crop Stress Biology for Arid Areas, College of Horticulture, Northwest A&F University, Yangling, 712100 Shaanxi China; 2 State Key Laboratory of Crop Stress Biology for Arid Areas, College of Plant Protection, Northwest A&F University, Yangling, 712100 Shaanxi China

**Keywords:** Plant virus, Preservation, *Apple stem grooving virus*, Cryopreservation

## Abstract

**Background:**

Preservation of plant virus is a fundamental requirement in all types of virus-related research and applied applications. Development of efficient, reliable strategies for long-term preservation of plant virus would largely assist these studies.

**Results:**

The present study reported a novel biotechnology allowing cryopreservation of Apple stem grooving virus (ASGV) in living shoot tips. Following cryopreservation by droplet-vitrification or encapsulation-dehydration, about 62–67% of shoot regrowth and 100% of ASGV cryopreservation were obtained. Although shoot proliferation and virus concentration were reduced in cryopreserved diseased shoots after 8 weeks of shoot regeneration, continuous subculture for 4 times (16 weeks) increased shoot proliferation and virus concentration to comparative levels as those produced by shoot tip culture (as a control to shoot tip cryopreservation). Cryopreserved ASGV was efficiently transmitted to a woody plant by micrografting and to a herbaceous indicator by mechanical inoculation. Gene sequencing in three fragments of ASGV genome including coat protein and movement protein showed that cryopreserved ASGV shared 99.87% nucleotide identities with shoot tip culture-preserved virus, indicating cryopreserved virus is genetically stable.

**Conclusions:**

The present study demonstrates ASGV, a representative virus that can infect meristematic cells of shoot tips, can be efficiently cryopreserved in shoot tips. To the best of our knowledge, this is the first report on plant virus cryopreservation in living tissues, and has great potential applications to long-term preservation of plant viruses.

**Electronic supplementary material:**

The online version of this article (10.1186/s13007-018-0312-9) contains supplementary material, which is available to authorized users.

## Background

Preservation of plant virus is a fundamental requirement in all types of virus-related research and applied applications such as antigen preparation for virus detection by immunology-based methods [1], production of vaccines [2, 3], genetic transformation to produce pathogen-derived resistant transgenic plants [3, 4] and bionanotechnology to produce nano drugs [5, 6]. Plant viruses are obligate intracellular parasites that replicate only inside the living cells of the hosts, and therefore cannot live without living tissues. In the past, various strategies have been developed for preservation of plant viruses, including freeze [7], freeze-drying [7–10], dehydration by physical drying [11] and chemicals [12], and in vitro culture [13, 14], among which freeze-drying was the most widely and reliable method. Although Cucumber mosaic virus lyophilized in the leaf or plant sap could be preserved for up to 240 days in the protecting medium containing 5% (w/v) sorbitol and 3.6% (w/v) dextran, their infection efficiency rapidly decreased as preservation time increased, with only 7% infection frequency obtained after 240 day of preservation [10]. Recently, Fan et al. [15] reported preservation of viral genomes in 700-y-old caribou feces from a subarctic ice patch. Their results indicate virus can be cryopreserved for decades. Obviously, continuous developments of more efficient, reliable methods for long-term virus preservation have been an attractive strategy in virology.

Cryopreservation, i.e. storage of living cells, tissues and organs in extra low temperature, usually that of the liquid nitrogen (LN), was first proposed with a motive for long-term preservation of plant genetic resources by Sakai [16], is at present time considered an ideal means for long-term preservation of plant genetic resources and has been widely applied to almost all economically important crops [17]. Cryopreservation has widely been reported for long-term preservation of animal and human viruses [18], but has only a few successes in plant viruses [19]. In the study of De Wijs and Suda-Bachmann [19], particles of Potato virus Y (PVY) and Watermelon mosaic virus (WMMV) were cryopreserved in LN for 22 months for the former and 32 months for the latter, without any decreases in the virus infectivity. These data indicate cryostorage of virus seems a very promising long-term preservation method for plant viruses.

Virus is unevenly distributed in plants: virus titer decreases as the distance increases from apical dome (AD), thus resulting in low virus titer or even virus free area in the very top parts of AD [20]. In shoot tip cryopreservation, only cells at the top layers of the AD and in the youngest leaf primordia (LP) are able to survive, while other cells are killed, following freezing in LN [17, 21, 22]. Thus, plants regenerated from cryopreserved shoot tips may be free of virus [17, 21, 22]. Based on this idea, shoot tip cryotherapy has been successfully established to efficiently eradicate plant pathogens including viruses [21–23]. It has been known some viruses and almost all viroids can infect meristematic cells in the shoot tips [24–27]. For these viruses and viroids, cryotherapy failed to eradicate them [28–30]. Based on these data, we hypothesize that shoot tip cryopreservation can be used for long-term preservation of the viruses that can infect meristematic cells of the shoot tips.

ASGV, a type member of the genus Capillovirus, is a single-stranded RNA virus, and the virus particles are flexuous filaments 620 nm long (apple strain), 650 nm long (citrus strain) and 680 nm long (*Actinidia* isolate), with obvious cross banding and helical symmetry [31]. ASGV is one of the most important latent viruses that infect *Malus* crops. It also attacks other fruit crops including *Pyrus*, *Citrus* and *Actinidia*, as well as a number of important ornamental crops [31]. ASGV can be transmitted by vegetative propagation like grafting and mechanical inoculation [31], and infect meristematic cells of the apical dome [30]. The objective of the present study was, therefore, to attempt to cryopreserve *Apple stem grooving virus* (ASGV) in shoot tips of ‘Gala’ apple. Cryopreserved virus was also tested for its infectious ability to infect apple (a woody plant) by micrografting and *Nicotiana benthamiana* (a herbaceous indicator) by mechanical inoculation. Gene fragments of cryopreserved ASGV genome were sequenced.

## Methods

### Plant material

‘Gala’ apple in vitro healthy and ASGV-infected shoots, which had been obtained by Wang et al. [32], were maintained as in vitro stock shoots on shoot maintenance medium (SMM) composed of Murashige and Skoog [33] medium (MS) supplemented with 1.1 µM 6-benzyladenine (BA), 0.05 µM indole-3-butyric acid (IBA), 30 g/l sucrose and 8 g/l agar [34]. The pH was adjusted to 5.8 prior to autoclaving at 121 °C for 20 min. All shoots were maintained at 22 ± 2 °C under a 16-h photoperiod at 50 µmol/m^2^ s provided by cool-white fluorescent tubes. Subculturing was performed every 4 weeks.

### Virus cryopreservation

Virus was cryopreserved in shoot tips by droplet-vitrification and encapsulation-dehydration, as described by Li et al. [35] and Feng et al. [36], respectively. Shoot tips (1.5 mm in size) containing 5–6 LPs excised from the diseased in vitro stock shoots were used for droplet-vitrification and encapsulation-dehydration. For droplet-vitrification [35], shoot tips were precultured for 1 day on precultured medium composed of semisolid MS supplemented with 2 M glycerol and 0.8 M sucrose. Precultured shoot tips were dehydrated at room temperature with PVS2 [MS supplemented with 30% (w/v) glycerol, 15% (w/v) ethylene glycol, 15% (w/v) dimethylsulfoxide and 0.4 M sucrose (pH 5.8)] [37] for 40 min, with 10 shoot tips per ml PVS2. After PVS2 treatment, each shoot tip was transferred into a droplet containing 2.5 µl PVS2 on aluminum foil strips (2 cm × 0.8 cm), and directly immersed in LN. After LN exposure for a few minutes, the foils with shoot tips were transferred into a 2-ml cryotube filled with LN for 1 h. Thawing was conducted by removing the frozen aluminum foil strips from LN and immediately placing into an unloading solution containing liquid MS supplemented with 1.2 M sucrose and incubated at room temperature for 20 min. For encapsulation-dehydration [36], shoot tips were encapsulated in Na-alginate beads (4–5 mm in diameter) and precultured with 0.5 M sucrose for 7 days, dehydrated in air in a laminar flow for 6 h to reduce bead moisture content to about 21% (fresh weight basis), prior to direct immersion in LN for cryopreservation for 1 h. Cryopreserved beads with shoot tips were rewarmed in a water bath at 38 °C for 2 min.

Shoot tips cryopreserved by the droplet-vitrification or encapsulation-dehydration procedure were post-thaw cultured on SMM in the dark for 3 days and then transferred to standard light conditions. Shoot tips (encapsulated and unencapsulated) were transferred after 12 and 36 h after the initial post-thaw culture to minimize oxidation reactions. Shoot tips cryopreserved by encapsulation-dehydration were extracted from the beads after 1 week of post-culture and then recovered directly on medium. Shoot regrowth was expressed as the percentage of surviving shoot-tips that formed normal shoots (≥5 mm in length) with at least three fully-opened leaves 8 weeks post-thaw culture. Subculture was performed once every 4 weeks. Regrown shoots (1.0 cm long) were transferred onto fresh SMM for shoot proliferation and subcultured once every 4 weeks. Shoot length and number of shoots proliferated per explant were measured in 1, 2 and 4 times of subculture.

Culture of shoot tips (the same size as used for shoot tip cryopreservation) excised from ASGV-infected shoots was used as control of virus cryopreservation. Shoot tips were cultured on SMM for shoot regrowth in the light condition, as described for in vitro stock shoots. Shoots regenerated after 8 weeks of culture were used for shoot proliferation, the same as for shoots regenerated from cryopreservation.

### Histological studies on micrografting, and virus transmission by micrografting and mechanical inoculation

**Fig. 1:**
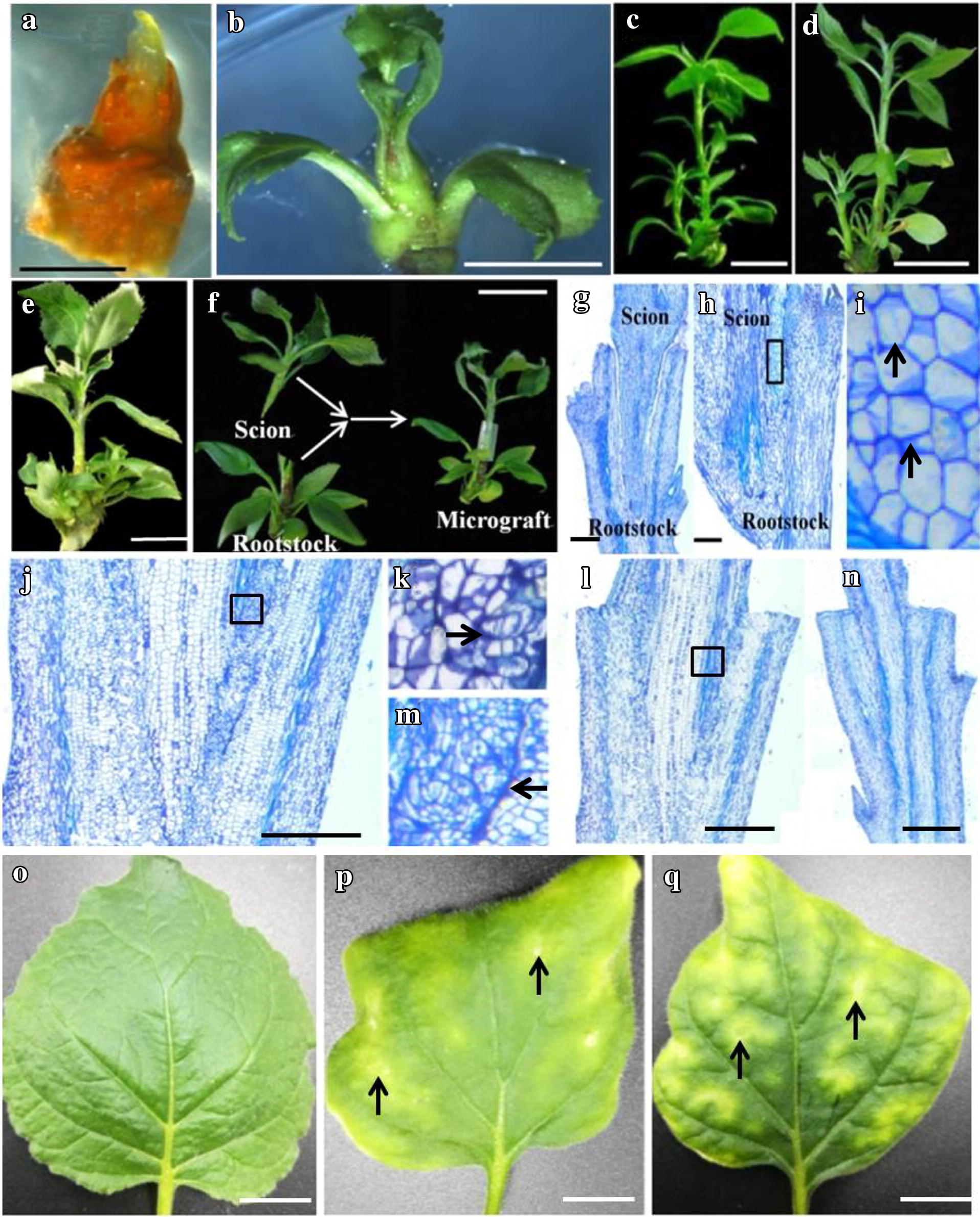
Survival and shoot regeneration of cryopreserved shoot tips of ASGV-infected shoots and histological studies on micrograft developments of shoots regenerated from cryopreservation for virus transmission in apple ‘Gala’. A surviving shoot tip (**a**), elongated shoot (**b**) and well-developed shoot (**c**) after 1, 8 and 16 weeks of post-culture following cryopreservation by droplet-vitrification. Shoot regenerated after 16 weeks of post-culture following cryopreservation by encapsulation-dehydration (**d**) and shoot tip culture (**e**). Preparations of scion and rootstock, and micrografting of the virus-infected shoots regenerated from cryopreserved shoot tips on the healthy rootstock (**f**). A longitudinal section of micrografts at day 0 of micrografting (**g**). A longitudinal section of micrografts at day 3 of micrografting (**h**). A closer view (**i**) showing callus formation, as indicated by arrows, in micrograft conjunction of scion and rootstock in black square in **h**. A longitudinal section of micrografts at day 7 of micrografting (**j**). A closer view (**k**) showing new cambial cells, as indicated by arrows, initiated from the callus formed in the micrograft conjunctions in black square in **j**. A longitudinal section of micrografts at day 10 of micrografting (**l**). A closer view (**m**) showing primary vascular bundle development, as indicated by arrows, in the micrograft conjunctions in black square in **l**. A longitudinal section of micrografts showing complete developments of vascular bundles between scion and rootstock at day 21 of micrografting (**n**). *N. benthamiana* leaves inoculated with sodium phosphate buffer without virus (**o**), with virus preserved by shoot tip culture (**p**) and with virus cryopreserved by droplet-vitrification (**q**). Typical ASGV symptoms are indicated by the black arrows. Bars in **a**, **c**–**f** and **o**–**q** = 1 cm; **b**, **g**, **h**, **j**, **l**, **n** = 1 mm

Shoots regenerated from cryopreserved shoot tips that had been subcultured for 4 times (16 weeks) were used for virus transmission by micrografting upon the healthy in vitro shoots of ‘Gala’ apple. Micrografting was conducted, as described by Hao et al. [38]. Shoot segments (1.0 cm long) with well-developed leaves were excised from middle to low parts of the diseased shoots regenerated from cryopreservation and shoot tip culture, and used as scions. The same size of shoot segments was excised from the healthy in vitro stock shoots of ‘Gala’ apple and used as rootstocks. A ‘V’ cut (about 0.5 cm in length) was made at the base of scions (Fig. 1f) and a vertical cut (about 0.5 cm in length) at the top of rootstocks (Fig. 1f). Micrografting was performed by inserting the ‘V’ shaped scions into the vertical cut of rootstocks, and then sterilized silicone tubes were used to hold the graft union (Fig. 1f). Micrografts were cultured on SMM and grown under the same culture conditions, as described for in vitro stock shoots, for 3 weeks.

Micrografts were taken at 0, 3, 7 and 14 days of micrografting and used for histological observations, as described by Hao et al. [38]. In brief, samples were fixed for 24 h in formalin-acetic-alcohol (FAA) (50% ethanol: formalin: acetic acid; 18:1:1), followed by dehydration in an ethanol series (50, 70, 85, 95 and 100%), with each concentration for 3 h, and finally stored in 100% ethanol until use. After embedding in paraffin, 5 μm thick samples were sectioned with a rotatory microtome (Leica RM 2235, Beijing, China) and stained with 0.05% toluidineblue. The stained sections were observed under a light microscope (Leica DM 2000). Micrograft success, which was defined as percentage of well-developed micrografts, was measured after 3 weeks of micrografting. Virus transmission was analyzed by RT-PCR, as described below, in virus status of the healthy rootstocks after 3, 7, 14 and 21 days of micrografting.

Seeds of *N. benthamiana* were sown in 8-cm pots containing peat (Pindstrup Substrate, Latvia) and grown for production of seedlings in a net-proof greenhouse under a 16-h photoperiod with a light intensity of 225 µmol/s m^2^ provided by Philips LED lamps at 24/20 °C of day/night temperatures. Virus transmission by mechanical inoculation was performed, according to Clover et al. [39]. In brief, fresh leaves (0.1 g) of the diseased shoots that had been subcultured for 4 times following cryopreservation were ground in 5 ml of 0.1 M sodium phosphate buffer (pH 7·5) made of 5% polyvinylpyrrolidone, 0.12% sodium sulphite and carbo-rundum powder to obtain ASGV. The third-fifth fully-opened leaves of 28-days old *N. benthamiana* seedlings were mechanically inoculated with ASGV. ASGV-induced symptoms were observed at day 21 of inoculation. Samples inoculated with only sodium phosphate buffer without virus were used as control.

### Analysis of ASGV by RT-PCR and RT-qPCR

Virus status was assessed by RT-PCR three times in the present study, according to Li et al. [30]. The first time was conducted in in vitro healthy and virus-infected shoots used for virus preservation by shoot tip cryopreservation and shoot tip culture. The second time was tested in shoots regenerated from cryopreserved shoot tips and shoot tip culture after 8 weeks of shoot regeneration. The third time was conducted in the healthy rootstocks micrografted by the virus-infected scions after 3, 7 and 14 days of micrografting. In brief, total RNA was extracted from fresh leaf tissue (0.5 g) using the Trizol Reagent (Invitrogen Ltd., Carlsbad, CA, USA), according to the manufacturer’s instructions. The cDNA was synthesized on 3 µg of total RNA using recombinant Moloney murine leukemia virus (M-MLV) reverse transcriptase (Promega, Madison, WI, USA), according to the manufacturer’s instructions. Random oligos were used for reverse transcription of mRNA in all samples. Forward and reverse primers (Additional file 1: Table S1) [40] were included in the PCR reaction, to amplify specific bands of 524 bp for ASGV. EF-1α was easily detected in all samples using the primers of EF-1α, confirming the procedure of cDNA synthesis was successful. The PCR products were separated by electrophoresis in 2% (w/v) agarose gel in Tris–acetate (TAE) buffer (40 mM Tris–acetate, 1 mM EDTA, pH 8.0), stained with 0.1% (w/v) ethidium bromide and visualized and photographed under ultraviolet light.

Relative mRNA expression levels of ASGV were analyzed by qRT-PCR in the diseased shoots regenerated from cryopreserved shoot tips after 1 (4 weeks), 2 (8 weeks), 4 (16 weeks) times of subculture of shoot proliferation. qRT-PCR was performed, according to Sun et al. [41]. Total RNA extraction and cDNA reverse transcription was performed as above. The qRT-PCR was performed using a CFX1000 (Bio-Rad, USA) instrument and a SYBR Premix ExTaq II Kit (Takara, Dalian, China) reagent. All primers used for qRT-PCR are given in Additional file 1: Table S1. Since EF-1α was stably expressed in samples, EF-1α was used as the reference gene. The relative mRNA expression levels of ASGV were expressed as Cq values.

### Gene fragment sequencing

ASGV cryopreserved by droplet-vitrification and preserved by shoot tip culture was used for gene fragment sequencing. Three gene fragments of ASGV genome with their nucleotides positioning from 235 to 756, 1210–2445 and 4310–6364 were cloned and sequenced, according to Zhao et al. [42]. Choice of these three fragments was because they locate in the front, middle and rear end, respectively, and the rear end contains coat protein (CP) and movement protein (MP). All primers used for gene fragment amplification are given in Additional file 1: Table S1. Total RNA extraction and cDNA reverse transcription was described as above in the analysis of ASGV by RT-PCR. The PCR conditions were: 94 °C for 3 min for pre-denaturation; 35 cycles of 94 °C for 30 s for denaturation, 52 °C for 30 s for primer annealing, 72 °C for 1 min per kb for extension; and 72 °C for 10 min for the final extension. The amplified fragments were purified and extracted using Gel Extraction Kit (BioTeke, Beijing, China). The purified DNA fragments were cloned into pMD19-T vector (TaKaRa Biotechnology Co., Dalian, China), and *Escherichia coli* strain JM109 was transformed. For each fragment, five clones were sequenced in both orientations to confirm the consensus sequence using an Applied Biosystems 3730 DNA Analyzer (Applied Biosystems, USA). Where polymorphic was encounter, at least five more clones were sequenced to obtain the consensus sequence. Sequence alignment analysis was performed using the program Vector NTI 11.5.1 (Invitrogen, USA).

### Experimental design and data analysis

In experiments of cryopreservation, shoot proliferation and micrografting, 10 samples were included in each treatment of three replicates. The whole experiment was repeated three times. Thus, each experiment had 30 samples in three replicates. Data are presented as mean ± SE. Statistical differences among means were assessed by analysis of variance (ANOVA) and significant difference (P < 0.05) was analyzed by Tukey’s multiple range test. For virus detection by RT-PCR and RT-qPCR, 20 samples randomly selected from the three independent experiments were included in each treatment. Ten samples from each treatment were used for gene fragment sequencing.

## Results

### Shoot regrowth, ASGV preservation and proliferation of virus-infected shoots following cryopreservation

Surviving shoot tips started to show green color at about 7 day of post-thaw culture following cryopreservation (Fig. 1a). Surviving shoot tips started to elongate and regrew into shoots with at least 3 fully-opened leaves after 8 weeks of post-thaw culture (Fig. 1b). Shoot regrowth rate was significantly higher (100%) in shoot tip culture than in droplet-vitrification and encapsulation-dehydration cryopreservation and 100% of ASGV were preserved by droplet-vitrification and encapsulation-dehydration cryopreservation, and shoot tip culture (Table 1). Shoot number and length were similar in ASGV-infected shoots regenerated from droplet-vitrification and encapsulation-dehydration cryopreservation, which were significantly lower in ASGV-infected shoots regenerated from shoot tip culture in the first and second subculture (Table 2). However, no differences were found in shoot number and length in ASGV-infected shoots regenerated from droplet-vitrification and encapsulation-dehydration cryopreservation, and shoot tip culture after the fourth time of subculture (Table 2, Fig. 1c–e).

**Table 1:** Effects of two cryopreservation methods on shoot regrowth and ASGV preservation of in vitro virus-infected shoots of apple ‘Gala’

Treatment	Shoot regrowth (%)	ASGV preservation (%)
Drop-vitri	67 ± 6b	100 (20/20)
Encap-dehy	62 ± 5b	100 (20/20)
Shoot tip culture	100a	100 (20/20)

Data of shoot regrowth and ASGV preservation were measured after 8 weeks of post-culture following cryopreservation. Results of shoot regrowth are presented as mean ± SE and with different letter in the same column indicating significant differences at *P *< 0.05. Numbers in parenthesis are number of samples showing positive response to RT-PCR/total number of samples tested

*Drop-vitri* droplet-vitrification, *Encap-dehy* encapsulation-dehydration

**Table 2:** Shoot proliferation of ASGV-infected apple ‘Gala’ shoots regenerated from cryopreservation and shoot tip culture in different times of subculture

Treatment	No. of shoots per explant			Mean shoot length (mm)		
	1*	2	4	1	2	4
Drop-vitri	0a	1.1 ± 0.2a	2.5 ± 0.4a	11.8 ± 0.5a	18.8 ± 1.1a	26.8 ± 2.4a
Encap-dehy	0a	1.1 ± 0.2a	2.5 ± 0.5a	12.8 ± 0.7a	19.8 ± 1.5a	26.8 ± 2.4a
Shoot tip culture	2.6 ± 0.5b	2.8 ± 0.4b	2.6 ± 0.5a	27.2 ± 2.1b	28.1 ± 2.2b	27.3 ± 2.2a

Data are presented as mean ± SE and with different letter in the same column indicating significant differences at *P *< 0.05

*Drop-vitri* droplet-vitrification, *Encap-dehy* encapsulation-dehydration

*Numbers 1, 2 and 4 represent 1, 2 and 4 times of subculture, respectively

### ASGV detection by RT-PCR and RT-qPCR

With RT-PCR, amplified segments of 524 bp were detected in in vitro diseased stock shoots that were used in the experiments for ASGV cryopreservation, while no such bands were detected in the healthy samples (Additional file 1: Fig. S1). Virus status in shoots regenerated from cryopreservation and shoot tip culture after 8 weeks of shoot regrowth was detected. All samples tested showed such specific bands, indicating ASGV was preserved (Additional file 1: Fig. S1). When RT-PCR was applied to test virus status in the healthy rootstocks micrografted by the infected scions, only samples showing 524 bp bands were considered infected (Additional file 1: Fig. S1).

Stable and similar values were obtained in the virus-infected shoots regenerated from cryopreservation and shoot tip culture when the reference gene *EF*-*1α* was used, indicating the RT-qPCR method used here was reliable. Virus concentration was much lower in ASGV-infected shoots regenerated from droplet-vitrification and encapsulation-dehydration cryopreservation than in shoot tip culture after the first and second subculture (Table 3). However, similar levels of virus concentrations were found in ASGV-infected shoots regenerated from droplet-vitrification and encapsulation-dehydration cryopreservation, and shoot tip culture after the fourth time of subculture (Table 3).

**Table 3:** Relative mRNA expressions levels (Cq values) of ASGV by qRT-PCR in the virus-infected shoots regenerated from cryopreservation of the virus-infected shoots of ‘Gala’ apple after different subculture times

Treatment	Subculture times after shoot regrowth					
	1*		2		4	
	ASGV	*EF*-*1α*	ASGV	*EF*-*1α*	ASGV	*EF*-*1α*
Drop-vitri	32.3 ± 0.4a	15.2 ± 0.8x	28.1 ± 0.6a	15.7 ± 0.4x	21.4 ± 0.5a	15.5 ± 0.3x
Encap-dehy	31.6 ± 0.6a	15.8 ± 0.7x	29.1 ± 0.3a	15.1 ± 0.4x	21.8 ± 0.6a	16.1 ± 0.4x
Shoot tip culture	26.2 ± 0.6b	16.3 ± .04x	22.5 ± 0.6b	15.2 ± 0.5x	20.2 ± 0.5a	15.1 ± 0.5x

Data are expressed in mean ± SE and with different letters indicating significant differences at *P *< 0.05. *EF*-*1α* gene was used as reference gene

*Drop-vitri* droplet-vitrification, *Encap-dehy* encapsulation-dehydration

*Numbers 1, 2 and 4 represent 1, 2 and 4 times of subculture, respectively

### Gene fragment sequencing

For the gene fragment A (Additional file 1: Fig. S2), one out of 522 nucleotides in cryopreserved virus was different from that in shoot tip culture-preserved virus, accounting for 0.2% of the total nucleotides (Additional file 1: Fig. S3A). No variations were found in nucleotides of the gene fragment B (Additional file 1: Fig. S2) between cryopreserved virus and shoot tip culture-preserved virus (Additional file 1: Fig. S3B). For the gene fragment C (Additional file 1: Fig. S2), four out of 2055 nucleotides in cryopreserved virus were different from those in shoot tip culture-preserved virus, accounting for 0.2% of the total nucleotides (Additional file 1: Fig. S3C). Taking together, five out of 3813 nucleotides of the three gene fragments sequenced in cryopreserved virus were detected different from those in shoot tip culture-preserved virus, accounting for 0.13% variations and 99.87% nucleotide identities.

### Histological studies on micrograft developments

With the help of silicone tubes (Fig. 1f), cut surface of the scions and rootstocks well contacted each other at day 0 of micrografting (Fig. 1g). Callus initiated in micrograft interface between the scions and rootstocks at day 3 of micrografting (Fig. 1h, i). New cambial cells initiated from the callus formed in the micrograft conjunctions at day 7 (Fig. 1j, k). Primary procambium and xylem started to develop from the new cambial cells at day 14 of micrografting (Fig. 1l, m) and these tissues continued to develop into vascular bundles connecting the scions and rootstocks at day 21 of micrografting (Fig. 1n).

### ASGV transmission by micrografting and mechanical inoculation

ASGV transmission by micrografting was identical between cryopreserved virus and shoot tip culture preserved virus at the different time points of micrografting. Virus was not detected in the healthy rootstocks at day 3 of micrografting (Table 4). Virus infection frequencies in the healthy rootstocks were 30, 60–70 and 100% at day 7, 14 and 21 of micrografting, respectively (Table 4). No symptoms were shown in leaves of *N. benthamiana* inoculated without virus (Fig. 1o), while typical ASGV symptoms were observed in leaves of *N. benthamiana* after 21 weeks of inoculation using cryopreserved virus (Fig. 1p) and shoot tip culture-preserved virus (Fig. 1q).

**Table 4:** Micrograft success and virus transmission in micrografts of ASGV-infected shoots regenerated from cryopreservation and shoot tip culture in ‘Gala’ apple

Treatment	Success of micrografts (%)	Virus transmission (%) after different time points (day) of micrografting			
		3	7	14	21
Drop-vitri	95 ± 5a	0 (0/20)	30 (6/20)	70 (14/20)	100 (20/20)
Encap-vitri	95 ± 5a	0 (0/20)	30 (6/20)	60 (12/20)	100 (20/20)
Shoot tip culture	100 ± 5a	0 (0/20)	30 (6/20)	70 (14/20)	100 (20/20)

Data of success of micrografts are recorded after 21 days of micrografting. Results are presented as mean ± SE and with different letters indicating significances at *P *< 0.05. Numbers in parenthesis are number of samples showing positive response to RT-PCR/total number of samples tested

*Drop-vitri* droplet-vitrification, *Encap-vitri* encapsulation-vitrification

## Discussion

So far, the use of virus particles is the most reliable method established for plant virus preservation. Some viruses preserved in this way are not stable and their infection ability decreased as time durations of preservation increased [8, 10, 11, 43]. For example, infection frequencies of CMV preserved by freeze-drying were 95% and only 7% after 15 and 240 days of preservation, respectively [10]. Furthermore, preserved virus can be used only by mechanical transmission to the target hosts [7–12, 19, 43]. It is well-known a number of plant viruses cannot be transmitted to the other host by mechanical transmission [24, 25], this largely limiting applications of the virus preservation. In in vitro culture for virus preservation, virus-infected tissues have to be periodically subcultured [13, 14]. Subculture has risks of contamination, which may result in total loss of the stored materials. In addition, in vitro *culture* can be used only for medium-term virus preservation. In the present study, ASGV was successfully cryopreserved in living shoot tips by droplet-vitrification and encapsulation-dehydration methods, the most popular cryogenic procedures that have been applied to almost all important crops [17]. To the best of our knowledge, this is the first study reporting plant virus cryopreservation in living tissues.

Frequencies of shoot regeneration of cryopreserved shoot tips and virus cryopreservation, as well as virus concentration preserved in the samples, and proliferation of virus-infected shoots regenerated from cryopreserved shoot tips, are the important factors determining virus cryopreservation efficiency. In the present study, more than 62–67% of cryopreserved shoot tips regrew into shoots. Theoretically, the samples can be stored in LN for an indefinite duration and maintain their recover ability, once they survive freezing in LN [17, 44]. Previous studies showed that shoot regrowth levels maintained high in dormant buds that had been cryopreserved for 10 years in *Malus* [45]. In the present study, 100% of virus cryopreservation frequencies were obtained in the droplet-vitrification and encapsulation-dehydration. The present study found virus concentration was low in the virus-infected shoots regenerated from the two cryopreservation methods after the first time (4 weeks) of subculture, increased with subculture times and reached the same levels of those in shoot tip culture-preserved virus after 4 times (16 weeks) of subculture. Cryopreservation cannot eradicate the viruses that can infect meristematic cells of AD, such as *Raspberry bushy dwarf virus* (RBDV) [28], *Pelargonium flower break virus* (PFBV) [46], *Pelargonium line pattern virus* (PLPV) [46] and ASGV in the present study. As stated above in Introduction section, only cells locating at the top layers of AD and in the youngest LPs were able to survive, while other cells were damaged or killed following cryopreservation [21–23]. Obviously, cryopreservation reduced the number of the virus-infected cells in shoot tips, and thus decreased the virus concentration in the shoots regenerated from cryopreservation, as shown by Gallard et al. [46], who attempted to eradicate by cryopreservation PFBV and PLPV. We believe this is also the case in this study. Although shoot proliferation of the virus-infected shoots regenerated from cryopreserved shoot tips was lower than those from shoot tip culture after the first (4 weeks) and second (8 weeks) times of subculture, rates of shoot proliferation were similar in the virus-infected shoots regenerated from cryopreservation and shoot tip culture after the fourth (16 weeks) time of subculture. Since many steps involved in cryopreservation such as preculture with high sugar concentration, dehydration by air drying or plant vitrification solutions and freezing in LN all caused stress to the cells [23], samples following cryopreservation need some time to recovery their growth ability, thus resulting in a lag period of shoot proliferation [47]. Results reported here indicate virus cryopreservation methods established in the present study are highly efficient.

ASGV can be transmitted by grafting and mechanical inoculation [31]. In the present study, cryopreserved virus was transmitted to ‘Gala’ apple (a woody plant) by micrografting, and *N. benthamiana* (a herbaceous indicator) by mechanical inoculation. Histological observations on micrografting showed micrografts developed normally, as noted in micrografting of virus-infected *grapevine leafroll*-*associated virus*-*3* [38]. Virus transmission from the diseased-scions to the healthy rootstocks occurred as vascular tissues developed in the micrografting conjunction, with 100% of ASGV transmitted at day 21 of micrografting, when vascular bundles connecting the scions and rootstock completely developed. These results were similar to those reported by Hao et al. [38], who used micrografting to transmit *Grapevine leafroll*-*associated virus*-*3*. Virus mechanical transmission showed typical symptoms of ASGV on the leaves of *N. benthamiana* after 21 days of inoculation. These results were consistent with those reported by De [48], Hirata et al. [49], Lovisolo et al. [50] and Bhardwaj et al. [51, 52] for mechanical transmission of ASGV from woody plants to herbaceous hosts. Our results indicate cryopreserved ASGV reported in the present study can be efficiently transmitted to infect healthy woody and herbaceous hosts.

Like plant germplasm, genetic stability is a major concern in virus preserved by any methods. When biological samples are frozen in LN, all cell division and metabolism are arrested, and the samples can be stored in LN, theoretically for an unlimited time period, thus limiting the opportunities for changes in the genetic integrity of stored materials [53, 54]. However, cryopreservation techniques involve not only storage in LN, but also other steps such as preculture with high sugar concentrations and dehydration by either physical drying or chemical solutions. All these factors may result in genetic variations of cryopreserved samples [17, 53, 54], especially in virus, which consists only of nucleic acids surrounded by protein coat. There have been a number of studies on assessments of genetic stability in the regenerants recovered from cryopreservation, and results so far obtained were quite promising [17, 53, 54]. Most of the studies on plant virus preservation tested the infection ability of the preserved virus [10, 13, 14] and however, information on genetic stability of preserved virus has been lacking. In the present study, we sequenced three gene fragments including CP and MP in cryopreserved virus, and found cryopreserved virus shared 99.87% nucleotide identities with shoot tip-preserved virus. These data indicated cryopreservation can maintain genetic stability of the stored virus.

Cryopreservation can eradicate viruses that are not able to infect meristematic cells of shoot tips and frequencies of virus eradication varied largely from 100% in *Sweet potato chlorotic stunt virus* (SPCSV) and Sweet potato feathery mottle virus (SPFMV) [55], to 30% in *Cucumber mosaic virus* (CMV) [56]. This means certain amounts of virus can be cryopreserved in shoot tips. Cell survival patterns, including number of surviving cells and location of surviving cells in AD, are critical for virus eradication frequency [28–30]. Cell survival patterns can be manipulated by time durations of preculture, types of plant vitrification solution, and cryogenic procedures [23, 35, 36, 57, 58]. Based on these facts, we speculate these manipulations can alter cell survival patterns, thus promoting frequency of cryopreserved viruses that are not able to infect meristematic cells of shoot tips. We are testing cryopreservation of other viruses that are not able to infect meristematic cells in the shoot tips.

In conclusion, the present study demonstrates ASGV, a representative virus that can infect meristematic cells of shoot tips, can be efficiently cryopreserved in shoot tips and cryopreserved ASGV can effectively be transmitted to woody plant by micrografting and to herbaceous indicator by mechanical inoculation. Cryopreserved ASGV is genetically stable. It is well-known that a number of viruses and almost all viroids can infect meristematic cells of shoot tips [24–27]. Thus, cryopreservation has great potential applications to long-term preservation of viruses and viroids.

## Additional file

**Additional file 1: Table S1.** Primers used for ASGV detection by RT-PCR, RT-qPCR and gene amplification in ASGV cryopreservation of virus-infected shoots of ‘Gala’ apple the present study. **Figure S1.** ASGV detection by RT-PCR for ASGV in in vitro stock shoots, and shoots regenerated from two cryopreservation methods and shoot tip culture in ‘Gala’ apple (A) and RT-PCR analysis for EF-1α gene as a reference (B). **Figure S2.** Gene fragments of ASGV genome used for gene sequencing in cryopreserved virus in in vitro shoots of ‘Gala’ apple. **Figure S3.** Comparison of gene fragments of A, B and C of ASGV genome preserved between cryopreservation and shoot tip culture.
