# Supplementary material for: Cryopreservation of virus: a novel biotechnology for long-term preservation of virus in shoot tips
Source: Plant Methods. 2018 Jun 12;14:47. doi: 10.1186/s13007-018-0312-9 (PMC5996562; doi:10.1186/s13007-018-0312-9)
Supplement: Supplementary file 1 — Additional file 1: Table S1. Primers used for ASGV detection by RT-PCR, RT-qPCR and gene amplification in ASGV cryopreservation of virus-infected shoots of ‘Gala’ apple the present study. Figure S1. ASGV detection by RT-PCR for ASGV in in vitro stock shoots, and shoots regenerated from two cryopreservation methods and shoot tip culture in ‘Gala’ apple (A) and RT-PCR analysis for EF-1α gene as a reference (B). Figure S2. Gene fragments of ASGV genome used for gene sequencing in cryopreserved virus in in vitro shoots of ‘Gala’ apple. Figure S3. Comparison of gene fragments of A, B and C of ASGV genome preserved between cryopreservation and shoot tip culture. [file 13007_2018_312_MOESM1_ESM.docx]

**Supplementary Material**

**Table S1** Primers used for ASGV detection by RT-PCR，RT-qPCR and gene amplification in ASGV cryopreservation of virus-infected shoots of ‘Gala’ apple the present study.

| Primers used for | Names of primers | Primer sequence | Ref. |
| --- | --- | --- | --- |
| RT-PCR | Forward | 5’-CTGCAAGACCGCGACCAAGTTT-3’ | [40] |
|  | Reverse | 5’-CCCGCTGTTGGATTTGATACACCTC-3’ |  |
| Gene amplification | F-235 | TCAGATCACATTCCCACCCAATATCCAA | Designed in the present study |
|  | R-756 | CGTAACATTCAGAGGCAACACCATCAG |  |
|  | F-1210 | TCATTGCTGCTAGTTTCTTTGA |  |
|  | R-2445 | TTCTTTTGACACTTGGTGTGTA |  |
|  | F-4310 | CAATACATTTGCGAATATGCTG |  |
|  | R-6364 | TGATACGACTTCTAACCCTCC |  |
| qRT-PCR | ASGVQF1 | GGAGACTGACGAAGGAAGGAAGG |  |
|  | ASGVQR1 | CGTTCAAAGAGTTCTGCCTGGAAG |  |
|  | EF-1α (F) | ATTCAAGTATGCCTGGGTGC | [41] |
|  | EF-1α (R) | CAGTCAGCCTGTGATGTTCC |  |

**
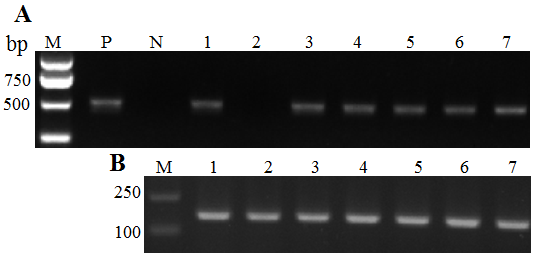
**

**Fig. S1** ASGV detection by RT-PCR for ASGV in *in vitro* stock shoots, and shoots regenerated from two cryopreservation methods and shoot tip culture in ‘Gala’ apple (A) and RT-PCR analysis for EF-1α gene as a reference (B). M=marker; P=positive control; N=negative control; Lane1=*in vitro* virus-infected stock shoots used for cryopreservation; Lane 2=*in vitro* healthy stock shoots; Lane 3=shoots regenerated from virus-infected shoot tips cryopreserved by droplet-vitrification; Lane 4=shoots regenerated from virus-infected shoot tips cryopreserved by encapsulation-dehydration; Lane 5=shoots regenerated from shoot tip culture of virus-infected shoots. Lanes 6-7=healthy rootstocks after 7 and 21 days of micrografting by the virus-infected scions.


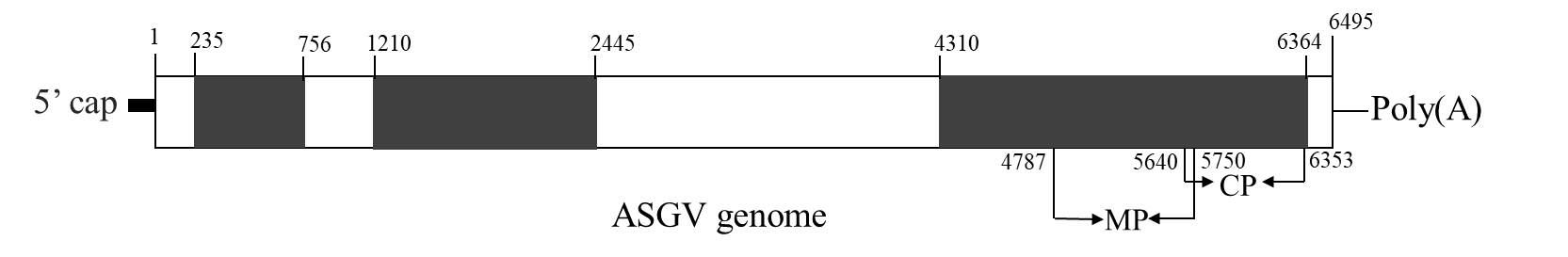


**B**

**A**

**C**

**Fig. S2** Gene fragments of ASGV genome used for gene sequencing in cryopreserved virus in *in vitro* shoots of ‘Gala apple. Gene fragment A contains nucleotides positioning at 235-756 (A). Gene fragment B contains nucleotides positioning at 1210-2445 (B). Gene fragment C contains nucleotides positioning at 4310-6364 including CP and MP of ASGV (C). The diagram of ASGV genome was drawn based on Liebenberg et al. [57].

**A**

| Nucleotide Position | **235** | **284** |
| --- | --- | --- |
| Cryopreservation | TCAGATCACATTCCCACCCAATATCCAAAATGATAGAGAACCATCTCTTG | |
| *In vitro* culture | TCAGATCACATTCCCACCCAATATCCAAAATGATAGAGAACCATCTCTTG | |
| Nucleotide Position | **285** | **334** |
| Cryopreservation | TATATATGTATTCCTAGCCTTTTGTCTTCTTTTAGGTCAGTTGCCTTCTT | |
| *In vitro* culture | TATATATGTATTCCTAGCCTTTTGTCTTCTTTTAGGTCAGTTGCCTTCTT | |
| Nucleotide Position | **335** | **384** |
| Cryopreservation | TTCTTTGAGAGAAAATAAAGTGAATAGTTTTTTGAAAATGCATTCAGTTT | |
| *In vitro* culture | TTCTTTGAGAGAAAATAAAGTGAATAGTTTTTTGAAAATGCATTCATTTT | |
| Nucleotide Position | **385** | **434** |
| Cryopreservation | TTTCACACGGAAAAATCAAATCTCTAGGCATGTACAACGCCATAATTGAT | |
| *In vitro* culture | TTTCACACGGAAAAATCAAATCTCTAGGCATGTACAACGCCATAATTGAT | |
| Nucleotide Position | **435** | 484 |
| Cryopreservation | GGGAAAGACAAATACAGGTATGGTGACGTCCCGTTTTCCTCTTTTAGGGA | |
| *In vitro* culture | GGGAAAGACAAATACAGGTATGGTGACGTCCCGTTTTCCTCTTTTAGGGA | |
| Nucleotide Position | **485** | **534** |
| Cryopreservation | CAGAGTTACTGGCCTGAGAGATGAATGCTTGACACGTAATAAATTTCCGA | |
| *In vitro* culture | CAGAGTTACTGGCCTGAGAGATGAATGCTTGACACGTAATAAATTTCCGA | |
| Nucleotide Position | **535** | **584** |
| Cryopreservation | AGGTCCTTTTTCTTCATGATGAGTTGCACTTTCTCAGTCCCTTCGATATT | |
| *In vitro* culture | AGGTCCTTTTTCTTCATGATGAGTTGCACTTTCTCAGTCCCTTCGATATT | |
| Nucleotide Position | **585** | **634** |
| Cryopreservation | GCTTTCTTGTTTGAGACTATCCCAGAAATCGATAGAGTTGTTGCAACCAC | |
| *In vitro* culture | GCTTTCTTGTTTGAGACTATCCCAGAAATCGATAGAGTTGTTGCAACCAC | |
| Nucleotide Position | **635** | **684** |
| Cryopreservation | AGTCTTTCCTATCGAACTTCTTTTTGGGGACAAGGTTTCAAAAGAGCCCA | |
| *In vitro* culture | AGTCTTTCCTATCGAACTTCTTTTTGGGGACAAGGTTTCAAAAGAGCCCA | |
| Nucleotide Position | **685** | **734** |
| Cryopreservation | GGGTCTACACTTACAAAGTTCATGGCTCTTCTTTCTCATTTTACCCTGAT | |
| *In vitro* culture | GGGTCTACACTTACAAAGTTCATGGCTCTTCTTTCTCATTTTACCCTGAT | |
| Nucleotide Position | **735** **756** |  |
| Cryopreservation | GGTGTTGCCTCTGAATGTTACG | |
| *In vitro* culture | GGTGTTGCCTCTGAATGTTACG | |

| **B**  Nucleotide Position | **1210** | **1259** |
| --- | --- | --- |
| Cryopreservation | TCATTGCTGCTAGTTTCTTTGAACCAACTGAATTTCATCTAAACATGAGG | |
| *In vitro* culture | TCATTGCTGCTAGTTTCTTTGAACCAACTGAATTTCATCTAAACATGAGG | |
| Nucleotide Position | **1260** | **1309** |
| Cryopreservation | AAGCTGTTAAGCGATCTTGCTACCAAAGGAATCGAGGTTCCTCTGGAAGT | |
| *In vitro* culture | AAGCTGTTAAGCGATCTTGCTACCAAAGGAATCGAGGTTCCTCTGGAAGT | |
| Nucleotide Position | **1310** | **1359** |
| Cryopreservation | GATAGTTCTGGATAAAGTGAATTTCATAGAAACCAGATTCCATGCCAGGA | |
| *In vitro* culture | GATAGTTCTGGATAAAGTGAATTTCATAGAAACCAGATTCCATGCCAGGA | |
| Nucleotide Position | **1360** | **1409** |
| Cryopreservation | TGTTTGATATAGCACAGGCCCTTGGAGTGAAGTTGGAATTACTTGGCCGC | |
| *In vitro* culture | TGTTTGATATAGCACAGGCCCTTGGAGTGAAGTTGGAATTACTTGGCCGC | |
| Nucleotide Position | **1410** | **1459** |
| Cryopreservation | AGGTTTGACTATGAAGCTGAAAGCGAGGAGTACTTCTCAGAAGGTGGCT | |
| *In vitro* culture | AGGTTTGACTATGAAGCTGAAAGCGAGGAGTACTTCTCAGAAGGTGGCT | |
| Nucleotide Position | **1460** | **1509** |
| Cryopreservation | TCTTTTCATGCCCTCCAAGACAAATCCAGACAGGAATTGGGTCTTGAATT | |
| *In vitro* culture | TCTTTTCATGCCCTCCAAGACAAATCCAGACAGGAATTGGGTCTTGAATT | |
| Nucleotide Position | **1510** | **1559** |
| Cryopreservation | CTGGTGCTCTAAAGGTTGATTACTCGAAGTTAGTCAGGACGAAAAGATTC | |
| In vitro culture | CTGGTGCTCTAAAGGTTGATTACTCGAAGTTAGTCAGGACGAAAAGATTC | |
| Nucleotide Position | **1560** | **1609** |
| Cryopreservation | AGGCTCAAGAGAGACTTTGTTGACCTCATACTCAAAGGGGAAACTCCTAG | |
| *In vitro* culture | AGGCTCAAGAGAGACTTTGTTGACCTCATACTCAAAGGGGAAACTCCTAG | |
| Nucleotide Position | **1610** | **1659** |
| Cryopreservation | GGTTCAGCTCTTCTTGGAATCAAATCCTGACTCCAAATCTCAAAGCAAAA | |
| *In vitro* culture | GGTTCAGCTCTTCTTGGAATCAAATCCTGACTCCAAATCTCAAAGCAAAA | |
| Nucleotide Position | **1660** | **1709** |
| Cryopreservation | GTGCTAATAGAGAAGAAAAGCAATGTTTGGAACAAAAAGAAGATCCCGAG | |
| *In vitro* culture | GTGCTAATAGAGAAGAAAAGCAATGTTTGGAACAAAAAGAAGATCCCGAG | |
| Nucleotide Position | **1710** | **1759** |
| Cryopreservation | GGTAGTTCAACATTTGAGTCATCGAGCACACCAATGTGCACTGAGGATAG | |
| *In vitro* culture | GGTAGTTCAACATTTGAGTCATCGAGCACACCAATGTGCACTGAGGATAG | |
| Nucleotide Position | **1760** | **1809** |
| Cryopreservation | ACAGGGTTTTGAAGGTTCACTTCCAATTGACTTAATTAATTCATTTGAGT | |
| *In vitro* culture | ACAGGGTTTTGAAGGTTCACTTCCAATTGACTTAATTAATTCATTTGAGT | |
| Nucleotide Position | **1810** | **1859** |
| Cryopreservation | ACAGGGTTTTGAAGGTTCACTTCCAATTGACTTAATTAATTCATTTGAGT | |
| *In vitro* culture | ACAGGGTTTTGAAGGTTCACTTCCAATTGACTTAATTAATTCATTTGAGT | |
| Nucleotide Position | **1860** | **1909** |
| Cryopreservation | AAGGCCATTTCAGCTCATCTGGGGATTGACACTCAGGACCTTCTGAACTT | |
| *In vitro* culture | AAGGCCATTTCAGCTCATCTGGGGATTGACACTCAGGACCTTCTGAACTT | |

| Nucleotide Position | **1910** | **1959** |
| --- | --- | --- |
| Cryopreservation | TTTAGTTAATGAAGACATTTCTGATGAGCTGCTTGACTGCATTGAGGAAG | |
| *In vitro* culture | TTTAGTTAATGAAGACATTTCTGATGAGCTGCTTGACTGCATTGAGGAAG | |
| Nucleotide Position | **1960** | **2009** |
| Cryopreservation | ACAAAGGTCTGTCACACGAAATGATTGAAGAAGTTCTGGTGACCAAAGGT | |
| *In vitro* culture | ACAAAGGTCTGTCACACGAAATGATTGAAGAAGTTCTGGTGACCAAAGGT | |
| Nucleotide Position | **2010** | **2059** |
| Cryopreservation | CTCTCAATGGTTTACACGTCTGACTTCAAAGAGATGGCGGTTCTCAACAG | |
| *In vitro* culture | CTCTCAATGGTTTACACGTCTGACTTCAAAGAGATGGCGGTTCTCAACAG | |
| Nucleotide Position | **2060** | **2109** |
| Cryopreservation | GAAATATGGAGTAAATGGGAAGATGTATTGTACCATCAAGGGTAATCACT | |
| *In vitro* culture | GAAATATGGAGTAAATGGGAAGATGTATTGTACCATCAAGGGTAATCACT | |
| Nucleotide Position | **2110** | **2159** |
| Cryopreservation | GCGAGCTAAGTTCAAAGGAATGTTTCATTAGGCTTCTAAAAGAAGGTGGT | |
| *In vitro* culture | GCGAGCTAAGTTCAAAGGAATGTTTCATTAGGCTTCTAAAAGAAGGTGGT | |
| Nucleotide Position | **2210** | **2259** |
| Cryopreservation | CGGACGATTTGTGCATAATAGGGAAAGAGCCGTCAAATTGGCGAAGTCCA | |
| *In vitro* culture | CGGACGATTTGTGCATAATAGGGAAAGAGCCGTCAAATTGGCGAAGTCCA | |
| Nucleotide Position | **2260** | **2309** |
| Cryopreservation | TGGCTAGAGGTACCACAGGTGTTTTGAGTGAATTTGACTCGATTTTTTGT | |
| *In vitro* culture | TGGCTAGAGGTACCACAGGTGTTTTGAGTGAATTTGACTCGATTTTTTGT | |
| Nucleotide Position | **2310** | **2359** |
| Cryopreservation | AAAAGCATGGTTACTCTTTCAGAGCTGTTTCCTGAAAATTTTTCATCCAT | |
| *In vitro* culture | AAAAGCATGGTTACTCTTTCAGAGCTGTTTCCTGAAAATTTTTCATCCAT | |
| Nucleotide Position | **2360** | **2409** |
| Cryopreservation | AGTCGGATTGAGGTTGGGATTCGCTGGCTCAGGTAAGACCCACAAAGTTC | |
| *In vitro* culture | AGTCGGATTGAGGTTGGGATTCGCTGGCTCAGGTAAGACCCACAAAGTTC | |
| Nucleotide Position | **2410 2445** |  |
| Cryopreservation | TCCAATGGATCAACTACACACCAAGTGTCAAAAGAA | |
| *In vitro* culture | TCCAATGGATCAACTACACACCAAGTGTCAAAAGAA | |

| **C**  Nucleotide Position | **4310** | **4359** |
| --- | --- | --- |
| Cryopreservation | CAATACATTTGCGAATATGCTGTTTACAAAGTTGAAGTACAAGATAGATC | |
| *In vitro* culture | CAATACATTTGCGAATATGCTGTTTACAAAGTTGAAGTACAAGATAGATC | |
| Nucleotide Position | **4360** | **4409** |
| Cryopreservation | CAAGGAAACATAGAATACTTTTCGCTGGAGACGATATGTGTTCCCTGAGC | |
| *In vitro* culture | CAAGGAAACATAGAATACTTTTCGCTGGAGACGATATGTGTTCCCTGAGC | |
| Nucleotide Position | **4410** | **4459** |
| Cryopreservation | TCTTTGAAGAGAAGACGAAGTGAAAGATCAACGAGGCTATTGAAAAGCTT | |
| *In vitro* culture | TCTTTGAAGAGAAGACGAAGTGAAAGATCAACGAGGCTATTGAAAAGCTT | |
| Nucleotide Position | **4460** | **4509** |
| Cryopreservation | TTCTCTGACTGCTGTGGAAGAGGTCAGGAAATTCCCCATGTTTTGTGGAT | |
| *In vitro* culture | TTCTTTGACTGCTGTGGAAGAGGTCAGGAAATTCCCCATGTTTTGTGGAT | |
| Nucleotide Position | **4510** | **4559** |
| Cryopreservation | GGTATTTAAGCCCCTATGGAATAATCAAGTCGCCAAAATTACTATGGGCA | |
| *In vitro* culture | GGTATTTAAGCCCCTATGGAATAATCAAGTCGCCAAAATTACTATGGGCA | |
| Nucleotide Position | **4560** | **4609** |
| Cryopreservation | AGAATTAAGATGATGAGTGAGAGACAGCTCCTCAAGGAGTGTGTGGACAA | |
| *In vitro* culture | AGAATTAAGATGATGAGTGAGAGACAGCTCCTCAAGGAGTGTGTGGACAA | |
| Nucleotide Position | **4610** | **4659** |
| Cryopreservation | CTACTTATTTGAGGCAATATTTGCCTACAGATTAGGTGAGAGGCTTTACA | |
| *In vitro* culture | CTACTTATTTGAGGCAATATTTGCCTACAGATTAGGTGAGAGGCTTTACA | |
| Nucleotide Position | **4660** | **4709** |
| Cryopreservation | CAATTTTGAAAGAAGAAGACTTTGAGTATCATTACCTGGTTATAAGATTT | |
| *In vitro* culture | CAATTTTGAAAGAAGAAGACTTTGAGTATCATTACCTGGTTATAAGATTT | |
| Nucleotide Position | **4710** | **4759** |
| Cryopreservation | TTTGTTAAAAACTCAAAGCTGCTGACTGGATTGAGCAAGAGCTTGATATT | |
| *In vitro* culture | TTTGTTAAAAACTCAAAGCTGCTGACTGGATTGAGCAAGAGCTTGATATT | |
| Nucleotide Position | **4760** | **4809** |
| Cryopreservation | TGAAATTGGAGAAGGGATTGGGTCAGAATGGCTATCGTCAATGTCAACCA | |
| *In vitro* culture | TGAAATTGGAGAAGGGATTGGGTCAGAATGGCTATCGTCAATGTCAACCA | |
| Nucleotide Position | **4810** | **4859** |
| Cryopreservation | TTTCCTCAAGGAGGTCGAATCCACAGACCTCAAAATTGATGCAATTTCAT | |
| *In vitro* culture | TTTCCTCAAGGAGGTCGAATCCACAGACCTCAAAATTGATGCAATTTCAT | |
| Nucleotide Position | **4860** | **4909** |
| Cryopreservation | CTTCAGAACTCTATAAAGATGCAACTTTCTTCAAACCGGATGTACTCAAT | |
| *In vitro* culture | CTTCAGAACTCTATAAAGATGCAACTTTCTTCAAACCGGATGTACTCAAT | |
| Nucleotide Position | **4910** | **4959** |
| Cryopreservation | TGCATCAAGAGATTTGAGTCCAATGTTAAAGTCTCATCAAGATCGGGTGA | |
| *In vitro* culture | TGCATCAAGAGATTTGAGTCCAATGTTAAAGTCTCATCAAGATCGGGTGA | |
| Nucleotide Position | **4960** | **5009** |
| Cryopreservation | CGGGCTCGTATTATCTGACTTCAAGCTTCTTGACGACACCGAAATTGACT | |
| *In vitro* culture | CGGGCTCGTATTATCTGACTTCAAGCTTCTTGACGACACCGAAATTGACT | |

| Nucleotide Position | **5010** | **5059** |
| --- | --- | --- |
| Cryopreservation | CAATCAGGAAAAAGAGTAATAAGTATAAATACTTGCATTATGGAGTCATT | |
| *In vitro* culture | CAATCAGGAAAAAGAGTAATAAGTATAAATACTTGCATTATGGAGTCATT | |
| Nucleotide Position | **5060** | **5109** |
| Cryopreservation | TTGGTTGGAATCAAAGCCATGCTCCCAAACTTTAGAGGAATGGAGGGGAG | |
| *In vitro* culture | TTGGTTGGAATCAAAGCCATGCTCCCAAACTTTAGAGGAATGGAGGGGAG | |
| Nucleotide Position | **5110** | **5159** |
| Cryopreservation | GGTCATTGTGTATGACGGTGCTTGTCTTGATCCAGAAAGGGGTCACATCT | |
| *In vitro* culture | GGTCATTGTGTATGACGGTGCTTGTCTTGATCCAGAAAGGGGTCACATCT | |
| Nucleotide Position | **5160** | **5209** |
| Cryopreservation | GTTCATATTTGTTTAAGTTTGAGTCTGATTGTTGTTACTTCGGGCTCCGA | |
| *In vitro* culture | GTTCATATTTGTTTAAGTTTGAGTCTGATTGTTGTTACTTCGGGCTCCGA | |
| Nucleotide Position | **5210** | **5259** |
| Cryopreservation | CCAGAACACTGTTTGTCAACAACTGACGCTAACTTGGCAAAGAGGTTTAG | |
| *In vitro* culture | CCAGAACACTGTTTGTCAACAACTGACGCTAACTTGGCAAAGAGGTTTAG | |
| Nucleotide Position | **5260** | **5309** |
| Cryopreservation | GTTTCGTGTGGATTTTGACTGCCCCCAGTACGAACAAGACACTGAGTTGT | |
| *In vitro* culture | GTTTCGTGTGGATTTTGACTGCCCCCAGTACGAACAAGACACTGAGTTGT | |
| Nucleotide Position | **5310** | **5359** |
| Cryopreservation | TTGCTCTGGACATTGGAGTTGCCTACAGGTGCGTCAATTCAGCAAGATTT | |
| *In vitro* culture | TTGCTCTGGACATTGGAGTTGCCTACAGGTGCGTCAATTCAGCAAGATTT | |
| Nucleotide Position | **5360** | **5409** |
| Cryopreservation | TTGGAAACCAAGACTGGTGATTCAGGATGGGCTTCACGAGCAATCAGTGG | |
| *In vitro* culture | TTGGAAACCAAGACTGGTGATTCAGGATGGGCTTCACAAGCAATCAGTGG | |
| Nucleotide Position | **5410** | **5459** |
| Cryopreservation | GTGTGAAGCTCTTAAATTTAATGAAGAAATCAAGATGGCCATCTTGGATC | |
| *In vitro* culture | GTGTGAAGCTCTTAAATTTAATGAAGAAATCAAGATGGCCATCTTGGATC | |
| Nucleotide Position | **5460** | **5509** |
| Cryopreservation | ACAAATCCCCACTTTTTCTGGAAGAAGGTGCACCAAATGTGCACATTGAA | |
| *In vitro* culture | ACAAATCCCCACTTTTTCTGGAAGAAGGTGCACCAAATGTGCACATTGAA | |
| Nucleotide Position | **5510** | **5559** |
| Cryopreservation | AAGAGGTTGTTTAGAGGTGATAAAGTTAGAAGGTCACGCTCTATTTCTGC | |
| *In vitro* culture | AAGAGGTTGTTTAGAGGTGATAAAGTTAGAAGGTCACGCTCTATTTCTGC | |
| Nucleotide Position | **5560** | **5609** |
| Cryopreservation | TAAAAGGGGACCAAACTCAAAGCTGCAGGAAAAGAGAGGATTTAGGTCCC | |
| *In vitro* culture | TAAAAGGGGACCAAACTCAAAGCTGCAGGAAAAGAGAGGATTTAGGTCCC | |
| Nucleotide Position | **5610** | **5659** |
| Cryopreservation | TCTCAGCTAGGATTGAAAGATTTGGAGAAAATGAGTTTGGAAGACGTGCT | |
| *In vitro* culture | TCTCAGCTAGGATTGAAAGATTTGGAGAAAATGAGTTTGGAAGACGTGCT | |
| Nucleotide Position | **5660** | **5709** |
| Cryopreservation | TCAACAAGCGAGACGCCACCGGGTAGGAGTATATCTGTGGAAGACACACA | |
| *In vitro* culture | TCAACAAGCGAGACGCCACCGGGTAGGAGTATATCTGTGGAAGACACACA | |

| Nucleotide Position | **5710** | **5759** |
| --- | --- | --- |
| Cryopreservation | TAGACCCGGCAAAGGAACTTCTGACAGTTCCTCCCCCTGAAGCATTTAAA | |
| *In vitro* culture | TAGACCCGGCAAAGGAACTTCTGACAGTTCCTCCCCCTGAAGCATTTAAA | |
| Nucleotide Position | **5760** | **5809** |
| Cryopreservation | GAAGGTGAAAGCTTTGAAGGCAGAGAGCTTTACCTTCTTCTCTGCAATCA | |
| *In vitro* culture | GAAGGTGAAAGCTTTGAAGGCAGAGAGCTTTACCTTCTTCTCTGCAATCA | |
| Nucleotide Position | **5810** | **5859** |
| Cryopreservation | TTACTGTAAATATTTATTTGGTAATATTGCTGTTTTCGGGTCGTCTGACA | |
| *In vitro* culture | TTACTGTAAATATTTATTTGGTAATATTGCTGTTTTCGGGTCGTCTGACA | |
| Nucleotide Position | **5860** | **5909** |
| Cryopreservation | AGACCCAGTTTCCTGCTGTTGGATTTGATACCCCTCCGGTTCATTACAAT | |
| *In vitro* culture | AGACCCAGTTTCCTGCTGTTGGATTTGATACCCCTCCGGTTCATTACAAT | |
| Nucleotide Position | **5910** | **5959** |
| Cryopreservation | CTGACAACGACCCCAAAAGAAGGGGAAACTAATGAAGAAAAGAAGGCCAG | |
| *In vitro* culture | CTGACAACGACCCCAAAAGAAGGGGAAACTAATGAAGAAAAGAAGGCCAG | |
| Nucleotide Position | **5960** | **6009** |
| Cryopreservation | AGAGGGTTCGTCTGGCGAAAAAACCAAGATTTGGAGGATCGACTTGTCAA | |
| *In vitro* culture | AGAGGGTTCGTCTGGCGAAAAAACCAAGATTTGGAGGATCGACTTGTCAA | |
| Nucleotide Position | **6010** | **6059** |
| Cryopreservation | ATGTTGTCCCCGAATTGAAAACCTTTGCTGCCACTTCTAGGCAGAACTCT | |
| *In vitro* culture | ATGTTGTCCCCGAATTGAAAACCTTTGCTGCCACTTCTAGGCAGAACTCT | |
| Nucleotide Position | **6060** | **6109** |
| Cryopreservation | TTGAACGAATGCACGTTCAGAAAGCTTTGTGAGCCTTTTGCTGATTCGGC | |
| *In vitro* culture | TTGAACGAATGCACGTTCAGAAAGCTTTGTGAGCCTTTTGCTGATTTGGC | |
| Nucleotide Position | **6110** | **6159** |
| Cryopreservation | TCGCGAATTTCTTCATGAGAGGTGGTCCAAAGGATTGGCCACCAACATTT | |
| *In vitro* culture | TCGCGAATTTCTTCATGAGAGGTGGTCCAAAGGATTGGCCACCAACATTT | |
| Nucleotide Position | **6160** | **6209** |
| Cryopreservation | ATAAGAAATGGCCCAAAGCTTTTGAAAAAAGCCCGTGGGTGGCGTTTGAC | |
| *In vitro* culture | ATAAGAAATGGCCCAAAGCTTTTGAAAAAAGCCCATGGGTGGCGTTTGAC | |
| Nucleotide Position | **6210** | **6259** |
| Cryopreservation | TTTGCCACCGGTCTGAAAATGAATCGTTTAACACCTGATGAAAAACAGGT | |
| *In vitro* culture | TTTGCCACCGGTCTGAAAATGAATCGTTTAACACCTGATGAAAAACAGGT | |
| Nucleotide Position | **6260** | **6309** |
| Cryopreservation | GATTGATAGAATGACTAAAAGGCTTTTTCGTACTGAAGGACAAAAAGGGG | |
| *In vitro* culture | GATTGATAGAATGACTAAAAGGCTTTTTCGTACTGAAGGACAAAAAGGGG | |
| Nucleotide Position | **6310** | **6359** |
| Cryopreservation | TTTTCGAGGCGGGTTCGGAGAGTAACTTGGAACTGGAGGGTTAGAAGTCG | |
| *In vitro* culture | TTTTCGAGGCGGGTTCGGAGAGTAACTTGGAACTGGAGGGTTAGAAGTCG | |
| Nucleotide Position | **6360 6364** |  |
| Cryopreservation | TATCA | |
| *In vitro* culture | TATCA | |

**Fig. S3** Comparison of gene fragments of A, B and C of ASGV genome preserved between cryopreservation and shoot tip culture. Nucleotides marked with gray and black color represent the conserved and altered nucleotides.
